# Feasibility and efficacy of lung ultrasound to investigate pulmonary complications in patients who developed postoperative Hypoxaemia-a prospective study

**DOI:** 10.1186/s12871-020-01123-6

**Published:** 2020-09-01

**Authors:** Chen Xie, Kai Sun, Yueyang You, Yue Ming, Xiaoling Yu, Lina Yu, Jiapeng Huang, Min Yan

**Affiliations:** 1 Department of Anesthesiology and Pain Medicine, the Second Affiliated Hospital, School of Medicine, Zhejiang University, Jiefang Road 88th, Hangzhou, 310016 People’s Republic of China; 2 Department of Anesthesiology & Perioperative Medicine, University of Louisville, Louisville, KY 40202 USA; 3 Department of Anesthesiology and Pain Medicine, Second Affiliated Hospital, Zhejiang University School of Medicine, 88 Jiefang Road, Hangzhou, 310009 NO China

**Keywords:** Lung ultrasound, Atelectasis, Pneumothorax, Pleural effusion, Thoracic computed tomography

## Abstract

**Background:**

Postoperative pulmonary complications (PPCs) and hypoxaemia are associated with morbidity and mortality. We aimed to evaluate the feasibility and efficacy of lung ultrasound (LUS) to diagnose PPCs in patients suffering from hypoxaemia after general anaesthesia and compare the results to those of thoracic computed tomography (CT).

**Methods:**

Adult patients who received general anaesthesia and suffered from hypoxaemia in the postanaesthesia care unit (PACU) were analysed. Hypoxaemia was defined as an oxygen saturation measured by pulse oximetry (SPO_2_) less than 92% for more than 30 s under ambient air conditions. LUS was performed by two trained anaesthesiologists once hypoxaemia occurred. After LUS examination, each patient was transported to the radiology department for thoracic CT scan within 1 h before returning to the ward.

**Results:**

From January 2019 to May 2019, 113 patients (61 men) undergoing abdominal surgery (45 patients, 39.8%), video-assisted thoracic surgery (31 patients, 27.4%), major orthopaedic surgery (17 patients, 15.0%), neurosurgery (10 patients, 8.8%) or other surgery (10 patients, 8.8%) were included. CT diagnosed 327 of 1356 lung zones as atelectasis, while LUS revealed atelectasis in 311 of the CT-confirmed zones. Pneumothorax was detected by CT scan in 75 quadrants, 72 of which were detected by LUS. Pleural effusion was diagnosed in 144 zones on CT scan, and LUS detected 131 of these zones. LUS was reliable in diagnosing atelectasis (sensitivity 98.0%, specificity 96.7% and diagnostic accuracy 97.2%), pneumothorax (sensitivity 90.0%, specificity 98.9% and diagnostic accuracy 96.7%) and pleural effusion (sensitivity 92.9%, specificity 96.0% and diagnostic accuracy 95.1%).

**Conclusions:**

Lung ultrasound is feasible, efficient and accurate in diagnosing different aetiologies of postoperative hypoxia in healthy-weight patients in the PACU.

**Trial registration:**

Current Controlled Trials NCT03802175, 2018/12/05, www.ClinicalTrials.gov

## Background

Postoperative pulmonary complications (PPCs), including atelectasis, pulmonary oedema, pneumonia, etc., are common, persistent and related to poor patient outcomes, medical costs, hospital readmissions and even significant mortality [1]. Hypoxaemia is mainly caused by atelectasis and occurs frequently in the immediate postoperative recovery course in both paediatric and adult patients; hypoxaemia is also associated with nausea, vomiting, postoperative cognitive dysfunction, surgical site infection, arrhythmias, prolonged hospital stay and death [2–7].

Rapid diagnosis and appropriate management must be made by the anaesthesiologist once hypoxia occurs postoperatively. The use of chest X-rays (CXR) is limited due to the disadvantage of poor quality [8]. Although thoracic computed tomography (CT) is considered the gold standard to elucidate the causes of hypoxia, radiation exposure and the need to transfer unstable patients make CT a less-than-ideal tool. Bedside lung ultrasound (LUS) has the advantages of sensitivity, accuracy, non-radiation, non-invasiveness, reproducibility and convenience. LUS has been validated for the diagnosis of atelectasis, pneumonia, pleural effusion and pneumothorax [9–14].

The aim of this study was to evaluate the feasibility and efficacy of LUS to diagnose PPCs in patients suffering from hypoxaemia after general anaesthesia in the postanaesthesia care unit (PACU) and compare LUS results with those of thoracic CT.

## Methods

### Patients

The study was approved by the review committee of the Second Affiliated Hospital of Zhejiang University (IR2018001133, 2018/12/05) and registered at ClinicalTrials.gov (NCT03802175) before patient enrolment. Informed consent was obtained from all patients. Adult patients who received general anaesthesia and suffered hypoxaemia in the PACU were included in this study. Postoperative hypoxaemia was defined as decreased oxygen saturation measured by pulse oximetry (SPO_2_) less than 92% for greater than 30 s while under ambient air conditions 20 min after extubation [15]. The exclusion criteria included the following: covered surgical dressings from open thoracic or breast surgery preventing ultrasound examination; body mass index (BMI) greater than 40 kg/m^2^; lack of cooperation due to cognitive dysfunction; residual muscle relaxants resulting in incomplete recovery of muscle strength (train-of-four stimulation, TOF < 0.9); respiratory forgetfulness from residual opioid; haemodynamic instability; anaemia; and significant bleeding, fever or hypothermia. In addition, patients were withdrawn if their SPO_2_ declined to 85% or less or if admission to the intensive care unit (ICU) occurred.

### Anaesthesia protocol

Before the induction of anaesthesia, all patients were preoxygenated with an inspiration oxygen fraction (FiO_2_) of 1.0 for 3 min. Anaesthesia was induced with midazolam 0.05–0.1 mg/kg, sufentanil 25–35 μg, etomidate 0.2–0.4 mg/kg and rocuronium 0.5–0.7 mg/kg. A proper double-lumen endotracheal tube was intubated to perform one-lung ventilation (OLV) during video-assisted thoracoscopic surgery (VATS), whereas a common tracheal tube was inserted for two-lung ventilation (TLV) during non-VATS. Continuous intravenous propofol, remifentanil and inhalational sevoflurane were utilized for anaesthesia maintenance after intubation. Supplemental cisatracurium was provided for adequate muscle relaxation when needed. Volume-controlled ventilation with tidal volume of 5–8 mL/kg (5–6 mL/kg for OLV and 6–8 mL/kg for TLV), respiratory rate (RR) of 12–15 breaths/min, FiO_2_ of 0.5–0.6 and positive end-expiratory pressure (PEEP) of 5 cm H_2_O was utilized to maintain an end-tidal carbon dioxide pressure (P_ET_CO_2_) between 35 and 45 mmHg and a peak airway pressure of less than 30 cmH_2_O (specific parameter was adjusted according to the type of surgery and patient’s condition). Depth of anaesthesia monitoring was completed by bispectral index (BIS) with an appropriate value of 40–60. Before closing the chest, each patient undergoing VATS received a recruitment manoeuvre (RM) by forcing sustained inspiration at the level of 30–40 cm H_2_O airway pressure for 10–20 s, and then OLV was converted to TLV until extubation. In addition, a chest tube was connected to a water-sealed bottle to provide drainage of any leaked air or fluid. Those undergoing non-VATS did not receive RM. All patients were transported to the PACU after the operation. Before extubation, the mechanical ventilations in the PACU was the same as that in the operating room. Extubation was performed when the following criteria were met: VT > 5 mL/kg, minimal RR of 11 breaths/min, haemodynamic stability (a maximum variation in mean arterial pressure and heart rate was 20% around the baseline value), normothermia, and TOF ≥0.9. Neostigmine (0.02 mg/kg) was used for the reversal of neuromuscular blocking before extubation. After extubation, the patient inhaled oxygen through a face mask at 3–6 L/min for approximately 15 min, and then the face masks were removed. Patients were supplemented with oxygen again through masks as temporary treatment if the SPO_2_ declined to less than 92%.

### Lung ultrasound examination

**Fig. 1:**
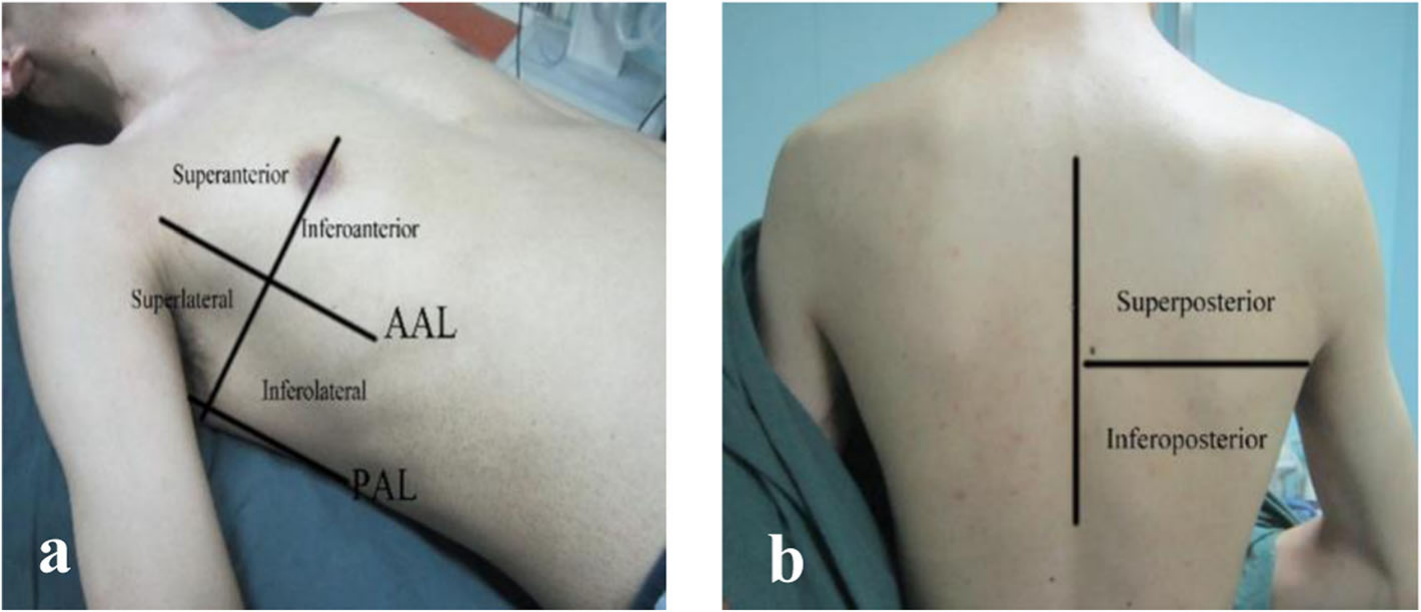
Hemithorax partition during lung ultrasound examination. **a**, **b** Each hemithorax was divided into 6 quadrants by anterior and posterior axillary lines. Abbreviations: AAL, anterior axillary line; PAL, posterior axillary line

With a 2 to 5 MHz convex probe in an ultrasound device (Mindray, Guangdong, China), LUS imaging was performed immediately in the PACU by two trained anaesthesiologists (Chen X and Kai S, both with more than 1 year of ultrasound training) once hypoxaemia occurred. The anterior and posterior axillary lines divided each hemithorax into three regions (anterior, lateral and posterior), and each region was further divided into two quadrants (superior and inferior) (Fig. 1). The anaesthesiologists performed LUS examination from the left lung to the right in the above order. Atelectasis was diagnosed as a tissue-like pattern or hypoechoic juxta-pleural consolidations with hyperechoic static air bronchograms [10]. A juxta-pleural consolidation or tissue-like structure may also indicate pneumonia. However, the visualization of dynamic air bronchograms helps exclude atelectasis [16]. With a negative predictive value of 100%, the presence of lung sliding excluded the diagnosis of pneumothorax [17]. Moreover, the diagnosis of pneumothorax should be combined with the lung point, barcode sign on M mode and absence of lung sliding [13, 18–20]. On this basis, the absence of pleural sliding in the anterior, lateral or posterior chest on LUS was defined as small, medium or large pneumothorax [21]. The presence of anechoic area fluctuating with respiration indicated pleural effusion [12]. Examination of pleural effusion was performed with the patient in the semi-recumbent position. A large pleural effusion was diagnosed when the maximal interpleural distance was more than 25 mm on ultrasonography, and the effusion must be visible in at least three intercostal spaces. Less than 15 mm of maximal interpleural distance was defined as a small effusion [22]. Combined with symptoms such as dyspnoea, a minimum of 3 B-lines in at least two anterior or lateral quadrants in each thorax may benefit from the consideration of pulmonary oedema [23].

**Fig. 2:**
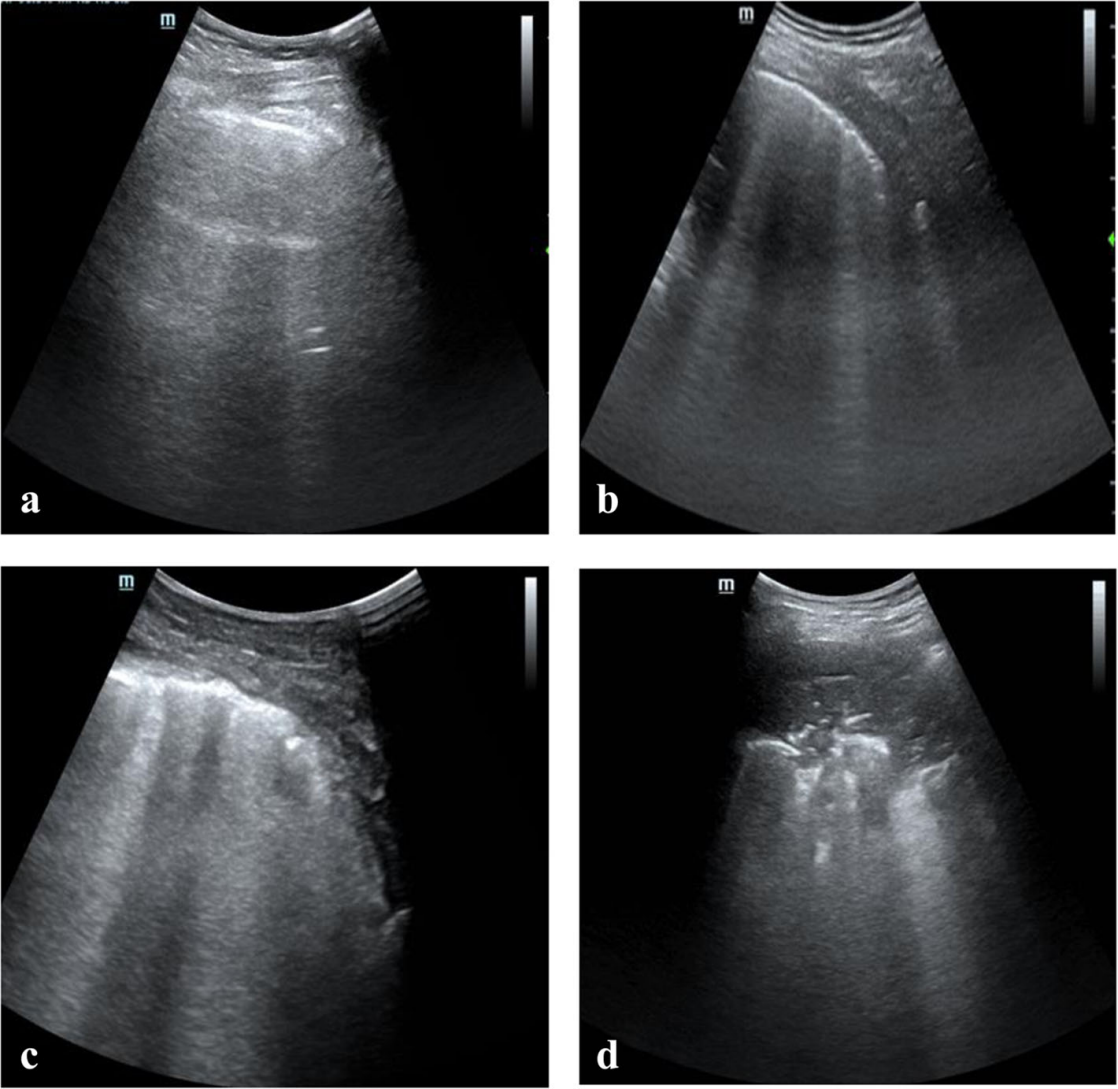
Lung ultrasound signs with different scores. **a** score 0, healthy lung, equidistant A-lines parallel to the sliding pleura; **b** score 1, moderate aeration loss, no fewer than 3 dispersive B lines originated from the pleura; **c** score 2, serious aeration loss, presence of coalescent B lines with irregular pleura; **d**, score 3, absolute aeration loss, subpleural consolidation

LUS scores (0–36, calculated by summing all 12 individual quadrant scores) are used to assess aeration changes, and a higher grade represents more serious aeration loss but is inapplicable for pneumothorax (Figure 2) [24–26]. The scoring system is as follows: score 0, healthy lung, equidistant A-lines parallel to the sliding pleura; score 1, moderate aeration loss, no fewer than 3 dispersive B lines originated from the pleural cavity; score 2, serious aeration loss, presence of coalescent B lines with pleural irregularities; and score 3, absolute aeration loss, subpleural consolidation. The stored video of the worst irregularity was analysed off-line by Chen X and Kai S. In case of disagreement, a third anaesthesiologist (Lina Y, with 5 years of ultrasound training) reviewed the uncertain images and made the final diagnosis.

### Computed tomography scan

After LUS examination, every patient with stable haemodynamic and spontaneous respiration was transported to the radiology department by a nurse anaesthetist for thoracic CT scan within 1 h after LUS examination. During transport, all patients received oxygen through face masks. Scanning from the apex to the diaphragm with the patient in the supine position, the examination was performed with a 128-slice spiral CT device (Siemens, Amberg, Germany). With a window width of 1500 Hounsfield units and a section thickness of 0.5 mm, all CT sections were stored for reconstruction and computerized analysis. A trained radiologist blinded to the study reported the CT findings by assessing the absence or presence of consolidation, effusion or pneumothorax as negative (−) or positive (+) in the same anatomic quadrant.

### Data collection

Demographic data, including sex, age, height, weight, American Society of Anesthetist (ASA) score, BMI, vital signs and smoking habit, were recorded. Medical history, pulmonary function test and physical examinations were extracted from the Electronic Medical Record. At the bedside, we collected surgical information, duration of mechanical ventilation and PACU stay, time needed for LUS examination and time needed for CT scan (transportation plus CT scan plus oral report). Cumulative opioid dose (calculated by duration and weight), volume of fluid administration (sum of crystalloid and colloid), blood products and arterial blood gas at the end of the operation, including haemoglobin, arterial partial pressure of oxygen (PaO_2_), and arterial partial pressure of carbon dioxide (PaCO_2_), were also recorded.

### Statistical analysis

PASS software (version 16.0) was used to calculate the sample size. Assuming the allowable error was 10% and α was 0.05 (bilateral), on the basis of a previous study, the estimated sensitivity and specificity of LUS were 87.7 and 92.1%, respectively [27]. The calculated sample sizes for sensitivity and specificity were 50 cases and 38 cases, respectively. Considering that the same sample size was adopted for both LUS examination and CT scan, 100 cases were taken from each group of 50 patients. The total sample size was 110 patients when considering a dropout rate of 10%. After testing the normality distribution, the mean ± standard deviation or median (interquartile range) was used to describe continuous variables, and comparisons were performed with a paired t test or Mann–Whitney U-test as appropriate. Categorical variables are expressed as frequencies and percentages and were compared with the chi-squared test or Fisher’s exact test. Spearman’s correlation coefficient was used to assess possible factors that may be associated with LUS scores. Correlation coefficient (r) values < 0.3 indicated nearly no correlation, r values between 0.3 and 0.5 indicated weak correlation, r values between 0.5 and 0.8 indicated moderate correlation and r values > 0.8 indicated a high level of correlation. Cohen’s kappa was used to test for agreement between the observers. Kappa equal to 0–0.20 indicated slight agreement, 0.21–0.40 indicated fair agreement, 0.41–0.60 indicated moderate agreement, 0.61–0.80 indicated substantial agreement, and 0.81–1 showed almost perfect agreement. SPSS statistical software version 23.0 (IBM Corp, Armonk, NY, USA) was used for data statistics and analysis.

## Results

**Fig. 3:**
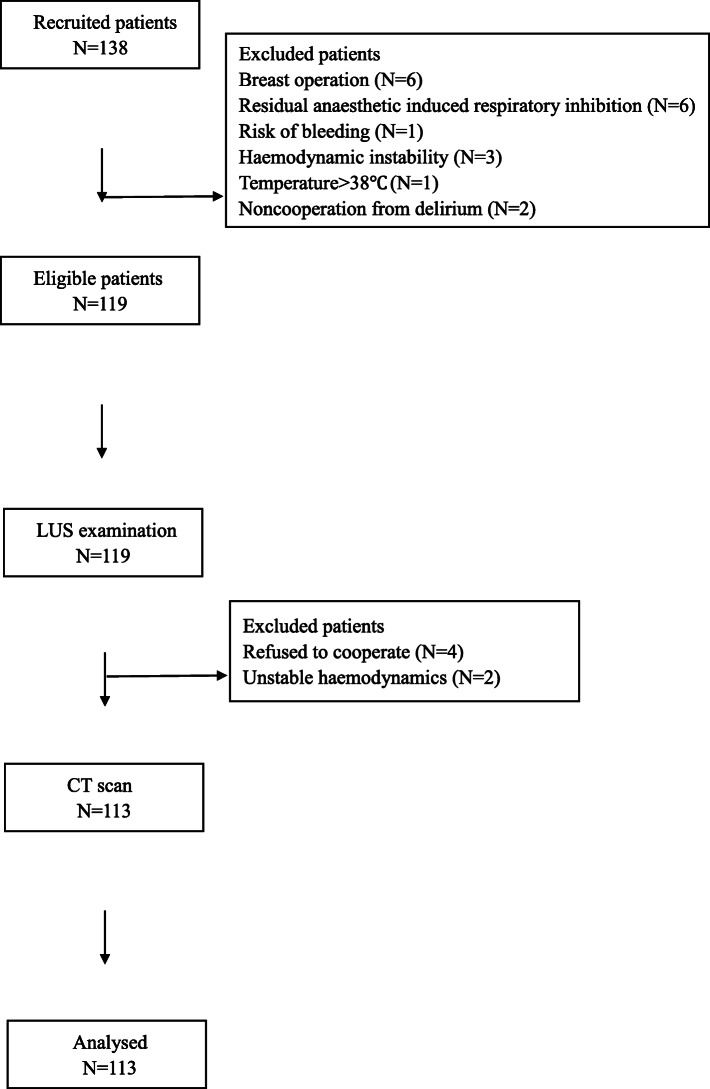
Flowchart of patient recruitment. Abbreviations: LUS, lung ultrasound; CT, computed tomography

From January to May 2019, 138 adult patients were evaluated for eligibility. Twenty-five patients (breast operation, haemodynamic instability, etc.) were excluded, and 113 patients were ultimately enrolled (Figure 3). During the study, all the LUS examinations and CT scans were performed successfully, and a total of 1356 pairs of ultrasound cine-loops and CT images were stored for all patients. Table 1 summarizes the demographic data of the enrolled patients.

**Table 1:** Patient Characteristics (*N* = 113)

Variables	Mean (SD)/median (IQR)
Age (y)	60.1 (12.0)
Sex, M/F(N)	61/52
Height (cm)	164.0 (7.5)
Weight (cm)	66.1 (10.8)
BMI (kg/m^2^)	24.5 (3.3)
ASA, 1/2/3 (N)	7/96/10
SPO_2_ (%)	90.0 (89.0, 91.0)
Duration of mechanical ventilation (min)	220.0 (162.5, 285.0)
Total infusion (mL)	1500.0 (1000.0, 1500.0)
Duration of PACU stay (min)	105.0 (85.0, 137.5)
LUS score	13.0 (11.0,16.0)
Smoking status, N (%)	
Current	10 (8.8)
Previous	11 (9.7)
Never	92 (81.4)
Type of surgery, N (%)	
Neurosurgery	10 (8.8)
Thoracoscopic surgery	31 (27.4)
Abdominal surgery	45 (39.8)
Major orthopaedics surgery	17 (15.0)
Others	10 (8.8)

Data are described as the mean ± standard deviation or median and interquartile range, as appropriate

The LUS score was described in patients without pneumothorax (*N* = 85)

SPO_2_ was assessed without inhaled oxygen 15 min after extubation

The duration of PACU stay was the time period from entering the PACU to leaving

Abbreviations: *SD* Standard deviation; *IQR* Interquartile range; *M* male; *F* Female; *BMI* Body mass index; *ASA* American Society of Anesthesiologists classification; *SPO*_*2*_ Oxygen saturation measured by pulse oximetry; *PACU* Postanaesthesia care unit; *LUS* Lung ultrasound

Postoperative hypoxaemia in the PACU mainly occurred in patients after abdominal surgery (45 patients, 39.8%) and VATS (31 patients, 27.4%), followed by major orthopaedic surgery (17 patients, 15.0%), neurosurgery (10 patients, 8.8%) and other types of surgery (10 patients, 8.8%). Eighty-two patients (72.6%) were diagnosed with atelectasis by both CT and LUS. CT scan diagnosed 327 of 1356 quadrants as atelectasis, while LUS revealed the same diagnosis in 311 of the 327 CT-diagnosed quadrants. In patients undergoing nonthoracic surgery, atelectasis was found to be in the posterior zones of both lungs, while the remaining atelectasis was discovered only in the operative lungs of VATS patients. Among the 82 patients with postoperative atelectasis, 19 patients showed signs of atelectasis on preoperative routine chest imaging examinations, while the remaining atelectasis was diagnosed only after surgery.

Twenty-eight patients (24.8%) (75 quadrants) were diagnosed with pneumothorax by CT scan, whereas 72 quadrants of these 75 quadrants were also diagnosed with pneumothorax with LUS. The majority of these pneumothorax patients (26 patients) were in the VATS group, and the pneumothorax was mainly distributed in anterior and lateral quadrants. In VATS patients, 11 cases of pneumothorax were small, while fifteen were medium in size. The other two patients underwent partial hepatectomy surgery. Regarding the last 2 patients, one was diagnosed with small pneumothorax both by LUS and CT scan, while another was diagnosed with medium pneumothorax, and CT reported an approximate 50% prevalence of tension pneumothorax.

Pleural effusion was found in 144 quadrants on CT scan in 39 patients (34.5%), which was primarily exhibited in posterior quadrants. LUS examination detected 131 quadrants with effusion among these CT-diagnosed zones. Nineteen patients (48.7%) received a diagnosis of pleural effusion on preoperative chest radiographs. The other 20 patients were newly diagnosed in the VATS group, all on the operative sides. One patient was diagnosed with massive pleural effusion on the left side, with visible anechoic effusion in the six quadrants.

One patient was diagnosed with diffuse interstitial syndrome due to multiple B-lines in all 12 lung quadrants, and a CT scan led to the same diagnosis. Both LUS examination and CT scan showed no abnormalities in 12 patients.

**Fig. 4:**
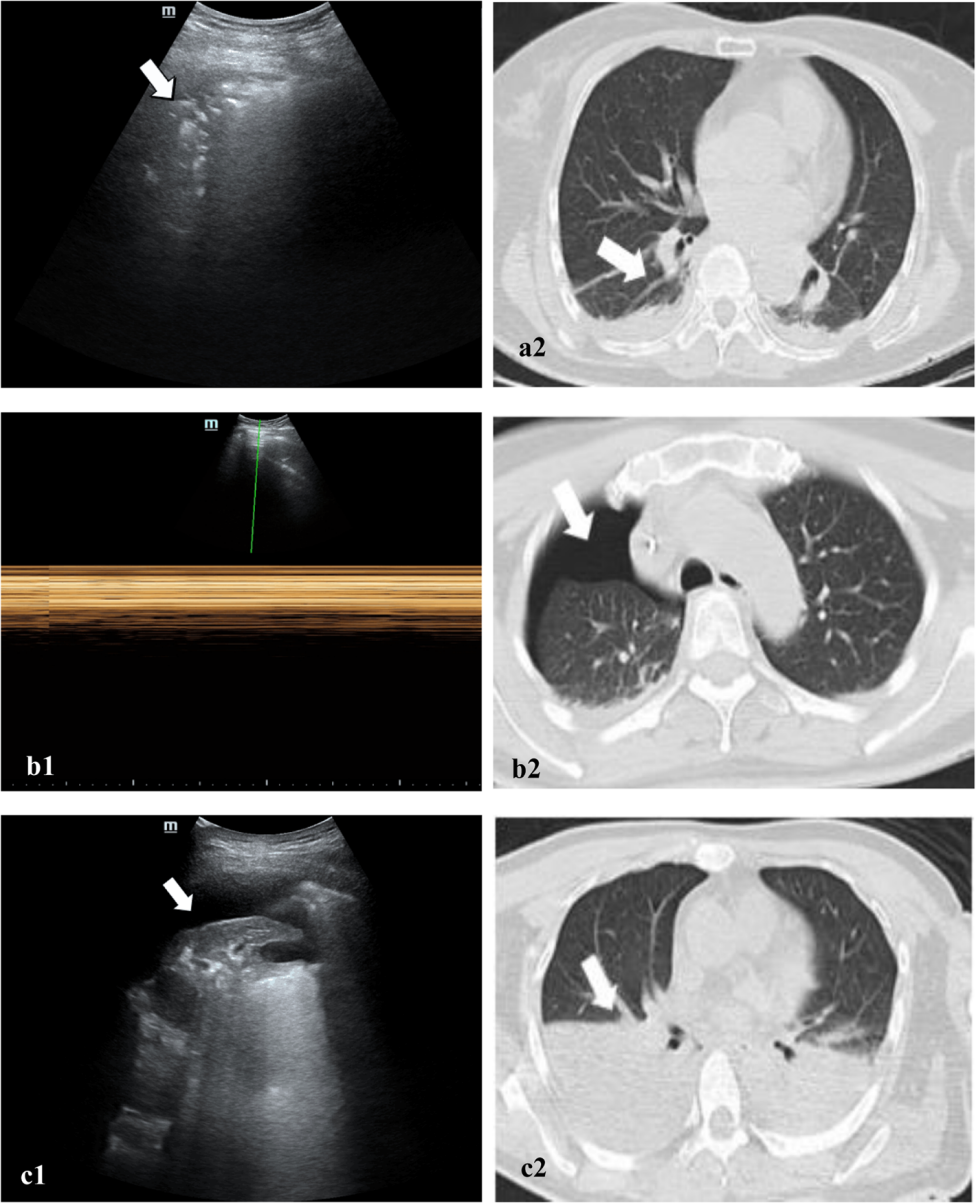
Typical pulmonary pathologies on both LUS and thoracic CT in the same regions. (**a**1) Typical LUS signs of atelectasis in the dorsal quadrant of the lung presented as tissue-like patterns (left, white arrow), (**a**2) CT signs of corresponding regions presented as a crescent shape (right, white arrow) (**b**1) Typical LUS on M-mode of pneumothorax in the anterior quadrant of the lung presented as a bar code sign, (**b**1) CT signs of corresponding regions presented as very-low-density gas window (right, white arrow). (**c**1) Typical LUS of pleural effusion in the dorsal quadrant of the lung presented as anechoic area (left, white arrow), (**c**2) CT signs of corresponding regions presented as a half-moon (right, white arrow). Abbreviations: LUS, lung ultrasound; CT, computed tomography.

The time needed for the LUS examination was significantly shorter than that needed for the CT scan (10.8 ± 1.8 min versus 26.8 ± 4.2 min, *P* < 0.001). Kappa values for the agreement between the first two observers of atelectasis, pneumothorax and pleural effusion were 0.951 (*P* < 0.001), 0.858 (*P* < 0.001) and 0.964 (*P* < 0.001), respectively. To resolve this disagreement, the third reviewer was mainly devoted to evaluating the diagnosis of pneumothorax. Table 2 shows the findings of LUS and CT scans for diagnosing atelectasis, pneumothorax and pleural effusion. LUS was reliable in the diagnosis of atelectasis (with a sensitivity of 98.0%, specificity of 96.7%, positive predictive value of 93.3%, negative predictive value of 99.1% and diagnostic accuracy of 97.2%), pneumothorax (with a sensitivity of 90.0%, specificity of 98.9%, positive predictive value of 96.0%, negative predictive value of 96.9% and diagnostic accuracy of 96.7%) and pleural effusion (sensitivity of 92.9%, specificity of 96.0%, positive predictive value of 91.0%, negative predictive value of 96.9% and diagnostic accuracy of 95.1%). Among the data we collected, post hoc analyses revealed no correlative factor that significantly influenced LUS scores (Table 3). Postoperative typical LUS and corresponding thoracic CT images of atelectasis, pneumothorax, and pleural effusion are displayed in Figure 4.

**Table 2:** Agreement between LUS and CT regarding pulmonary complications in accumulated quadrants

CT	LUS		Total
	+	–	
**a. Agreement between LUS and CT for atelectasis diagnosis**			
+	305	22	327
–	6	651	657
Total	311	673	984
**b. Agreement between LUS and CT for pneumothorax diagnosis**			
+	72	3	75
–	8	253	261
Total	80	256	336
**c. Agreement between LUS and CT for pleural effusion diagnosis**			
+	131	13	144
–	10	314	324
Total	141	327	468

**Table 3:** Correlation between possible factors and lung ultrasound scores (*N* = 85)

Variables	Correlation Coefficient (r)	*P*
Sex (M/F)	0.229	0.035
Age (y)	−0.041	0.707
BMI (kg/m^2^)	−0.127	0.246
SPO_2_ (%)	−0.244	0.024
Smoking	−0.039	0.725
Duration of mechanical ventilation (min)	−0.127	0.245
Type of surgery	0.075	0.494
Sufentanil dose (ug/kg/h)	0.125	0.253
Total infusion (mL)	0.046	0.677
Transfusion (Y/N)	−0.156	0.155

Lung ultrasound scores were recorded and analysed in patients without pneumothorax

Abbreviations: *LUS* Lung ultrasound; *M* Male; *F* Female; *BMI* Body mass index; *SPO*_*2*_ Oxygen saturation measured by pulse oximetry

## Discussion

Our study showed high accuracy of LUS in diagnosing PPCs such as atelectasis, pneumothorax and pleural effusion, with a high degree of sensitivity and specificity. Consistent with previous publications in both children and adults [28, 29], bedside LUS is reliable, portable, radiationless and fast for the investigation of pulmonary pathologic abnormalities. Previous publications on LUS were mostly from emergency departments and ICUs. To our knowledge, this is the first study to explore the application of LUS to investigate PPCs in patients who developed hypoxaemia in the PACU. In addition, our study population included patients undergoing various types of surgery, and patients with COPD or cardiovascular symptoms were not excluded. This may better reflect the real-world experience. Since postoperative thoracic CT is not routinely used, LUS in the PACU may help differentiate unexpected respiratory pathologies. Most likely, our study could provide clinical significance for the timely and appropriate treatment of postoperative hypoxaemia in the future.

PPCs are common after general anaesthesia, while hypoxaemia is primary triggered by atelectasis from compression, gas absorption and loss of surfactant [30]. Postoperative atelectasis was associated with pneumonia and could result in delayed discharge [31]. Early detection and treatment of atelectasis is essential for improving prognosis. Due to advantages such as simplicity, convenience, time requirement and non-radiation, LUS can be performed multiple times at the bedside. The sensitivity and specificity of the diagnosis of atelectasis by lung pulse in ultrasound were 93 and 100%, respectively [32, 33]. When compared with magnetic resonance imaging (MRI), LUS showed a sensitivity of 88%, specificity of 89% and accuracy of 88% in diagnosing pulmonary atelectasis [10]. LUS demonstrated excellent diagnostic accuracy (97.2%) in our study, which was higher than that reported (90.7%) by Yu X et al. [27] Yu’s study only enrolled patients undergoing elective intracranial surgery, and those with preoperative pulmonary comorbidities were excluded, whereas our study included a heterogeneous patient population for diversity. To eliminate the interference of the adipose layer in the ultrasonic image, obese patients (BMI > 40 kg/m^2^) were excluded. Considering the safety risk of transferring patients to CT scans, those with haemodynamic instability were also excluded. Hypoxaemia might also frequently occur in these patients, but the whole study only excluded 3 relevant patients (Figure 3), and this exclusion exerted almost no effect on the result. The incidence of postoperative atelectasis (67.2%) in our study was lower than that previously reported (90%) [34], which was partly due to routine recruitment manoeuvres at the end of the VATS group. Through lung-protective strategies such as low TV, a lower FiO_2_, higher RRRM and PEEP have been reported to significantly decrease postoperative respiratory complications [35–37] and were applied in our anaesthesia protocol. However, atelectasis still occurred frequently. PEEP has been reported as a successful method for improving oxygenation and respiratory function during general anaesthesia, but the optimal level is still inconclusive [38–40]. Although a PEEP of 5 cmH_2_O in our study has been reported in a previous study, a higher PEEP may be much more beneficial for reducing atelectasis formation, as has been recommended by some researchers [41]. RM combined with PEEP was also beneficial for reducing atelectrauma [42], but it was only performed in OLV in our study. This may explain the high occurrence of atelectasis in the non-VATS group. To date, the optimal systematization of RM remains a matter of debate, as findings have identified a potential danger of excessive RM during OLV, resulting in increased mortality [43]. Under perioperative ultrasound-guided recruitment manoeuvres and moderate PEEP, the incidence of atelectasis and postoperative hypoxaemia decreased in both infants and paediatric cardiac patients [41, 44, 45]. Detecting the effects of different levels of PEEP and RM on PPCs by lung ultrasound still needs more research.

Similar to Xirouchaki et al.’s findings, our study showed that LUS was effective in the diagnosis of pneumothorax [46]. The absence of lung sliding or B-line to diagnose pneumothorax by LUS has a sensitivity of 88 to 100% [47, 48]. Our study further confirmed the 90% sensitivity of LUS to diagnose pneumothorax. Because the thorax was opened for VATS, the pneumothorax was deemed residual gas. Patella et al. [47, 49] demonstrated that LUS could also effectively and accurately evaluate the small amount of pneumothorax remaining after thoracic drainage, which was faster and more accurate than CXR. Senniappan’s study suggested that LUS may be an alternative to CXR for the follow-up of pneumothorax due to its superior sensitivity, portability and reduction in radiation exposure [50]. Wei et al. [51] advocated for the use of daily LUS in the postoperative period to enhance recovery after thoracic surgery. Similar to prior publications [52–54], we demonstrated that LUS was sensitive and specific to diagnose pleural effusion with added benefits of convenience and safety. Compared with traditional methods, placing the thorax tube for fluid drainage under ultrasound guidance is safer and more effective and can reduce the incidence of pneumothorax [55]. The severity of hypoxaemia depended on effusion size and the patient’s cardiopulmonary condition, while prompt diagnosis of pleural effusion is vital to evaluate the optimal therapeutic choice. Effusion drainage ultrasound guidance would relieve compression atelectasis of the adjacent lung and improve respiratory mechanics and oxygenation.

Although LUS is an operator-dependent skill and adequate training is needed for effective clinical usage, it can be readily learned with a very simple device. See et al. [56] confirmed that after only 3 h of lung ultrasound self-study and an average of 15 scans of patients, the accuracy of diagnosis in trainees with no prior ultrasound experience would achieve 95.4%, and the medial scanning duration was only 12 min. In Zhan’s study, the implementer was a paediatric resident with no expert supervision and minimal practical ultrasound experience, and this examiner completed the LUS examinations accurately [11]. Compared to experts, inexperienced resident physicians in emergency medicine with 30 min LUS training can effectively identify B-lines with more than 80% sensitivity and specificity [57]. After brief training, surgical residents or medical students could also perform LUS well and interpret the results accurately [12]. To improve diagnostic reliability, ultrasound was performed and evaluated by two researchers with long-term training in our study. The results showed high agreement between the two observers, while the disagreement was mainly in regard to pneumothorax, probably because the diagnosis of pneumothorax by ultrasound should be based on multiple signs.

There are several limitations of our study. First, the 1-h time interval between LUS examination and CT scan and the suction impact of the water seal bottle may create false-positive results, while the obstruction of ultrasound views by the scapula and ribs could introduce false-negative results. Second, the presence of consolidation on LUS alone was insufficient for diagnosing pneumonia. In a recent study by Zhou et al. [58], the combination of LUS and procalcitonin had a better diagnostic value for pneumonia. Timely diagnosis of suspected aspiration pneumonia by LUS intraoperatively may be beneficial for patients but still needs more research in our future work. Third, different ventilatory management strategies may have different effects, especially on thoracic surgery, whereas the inclusion of multiple types of surgery patients was a limitation in our study. Although RM did not affect the accuracy of the comparison between LUS and CT, performing a subgroup analysis of this population might be meaningful. To refine our research, we conducted an LUS-related study specifically on thoracic surgery. Last, because of the BMI of the population in our study, our findings might not reflect the sensitivity and specificity of LUS to diagnose PPCs in patients with higher BMI, as those with a BMI > 40 kg/m^2^ were excluded.

## Conclusions

In conclusion, we showed that the application of LUS to diagnose the aetiologies of hypoxaemia in healthy-weight PACU patients is feasible and quick. LUS was sensitive and specific to diagnose PPCs when compared to the sensitivity and specificity of thoracic CT scans.

## Data Availability

The datasets generated and/or analysed during the current study are not publicly available due to the manuscript has not been received yet but are available from the corresponding author on reasonable request.

## Abbreviations

PPCs: Postoperative pulmonary complications
CRX: Chest x-rays
CT: Computed tomography
LUS: Lung ultrasound
PACU: Postanesthesia care unit
SPO_2_: Pulse oximetry
BMI: Body mass index
TOF: Train of four stimulation
ICU: Intensive care unit
FiO_2_: Inspiration oxygen fraction
OLV: One-lung ventilation
VATS: Video-assisted thoracoscopic surgery
TLV: Two-lung ventilation
RR: Respiratory rate
PEEP: Positive end-expiratory pressure
P_ET_CO_2_: End-tidal carbon dioxide pressure
BIS: Bispectral index
RM: Recruitment maneuver
ASA: American society of anesthetist
PaO_2_: Arterial partial pressure of oxygen
PaCO_2_: Arterial partial pressure of carbon dioxide
MRI: Magnetic resonance imaging
