# Correction to: Feasibility and efficacy of lung ultrasound to investigate pulmonary complications in patients who developed postoperative Hypoxaemia-a prospective study

**DOI:** 10.1186/s12871-020-01196-3

**Published:** 2020-11-09

**Authors:** Chen Xie, Kai Sun, Yueyang You, Yue Ming, Xiaoling Yu, Lina Yu, Jiapeng Huang, Min Yan

**Affiliations:** 1 Department of Anesthesiology and Pain Medicine, the Second Affiliated Hospital, School of Medicine, Zhejiang University, Jiefang Road 88th, Hangzhou, 310016 PR China; 2 Department of Anesthesiology & Perioperative Medicine, University of Louisville, Louisville, KY 40202 USA

**Correction to: BMC Anesthesiol 20, 220 (2020)**

**https://doi.org/10.1186/s12871-020-01123-6**

Following publication of the original article [1], the authors reported an error in Figs. 1 and 2 which are schematic figures of methods. The authors forgot to make relevant references to the figures as these have been published in authors previous work, Xie et al. 2020 [2].

Xie et al. 2020 [2] has been added to the captions of Figs. 1 and 2.

**Fig. 1:**
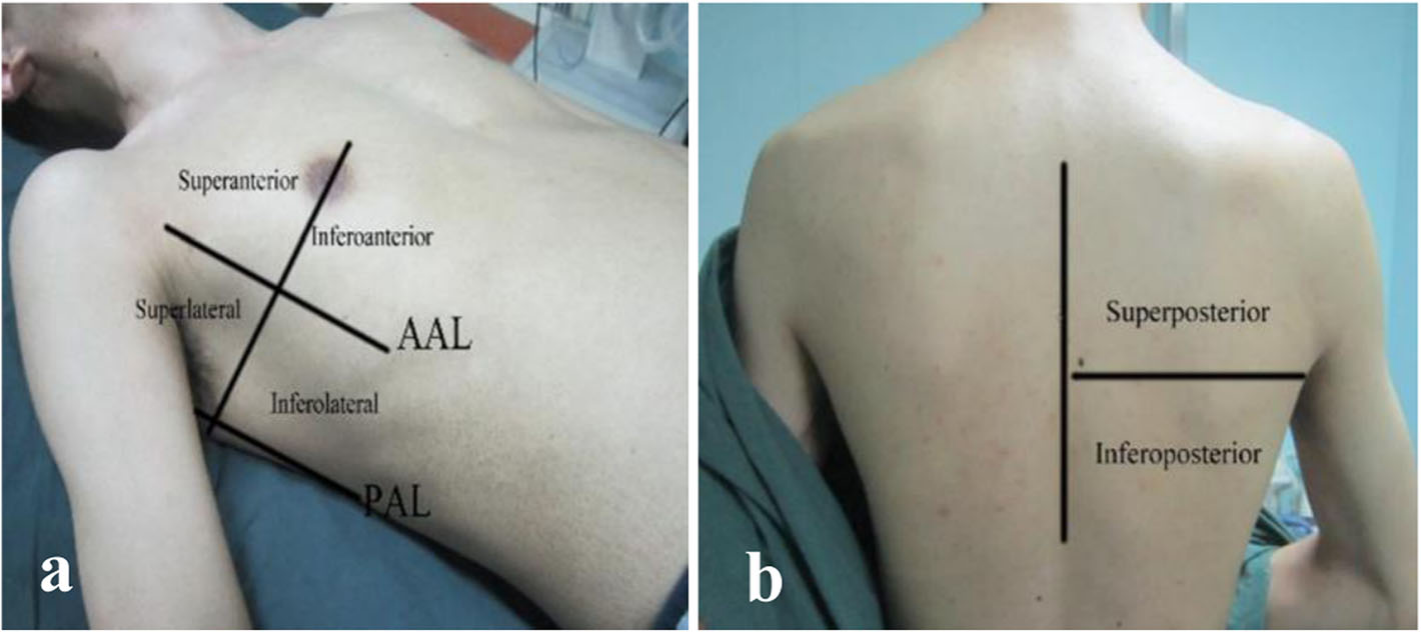
Hemithorax partition during lung ultrasound examination. **a**, **b** Each hemithorax was divided into 6 quadrants by anterior and posterior axillary lines. Abbreviations: AAL, anterior axillary line; PAL, posterior axillary line. Xie et al. 2020 [2]

**Fig. 2:**
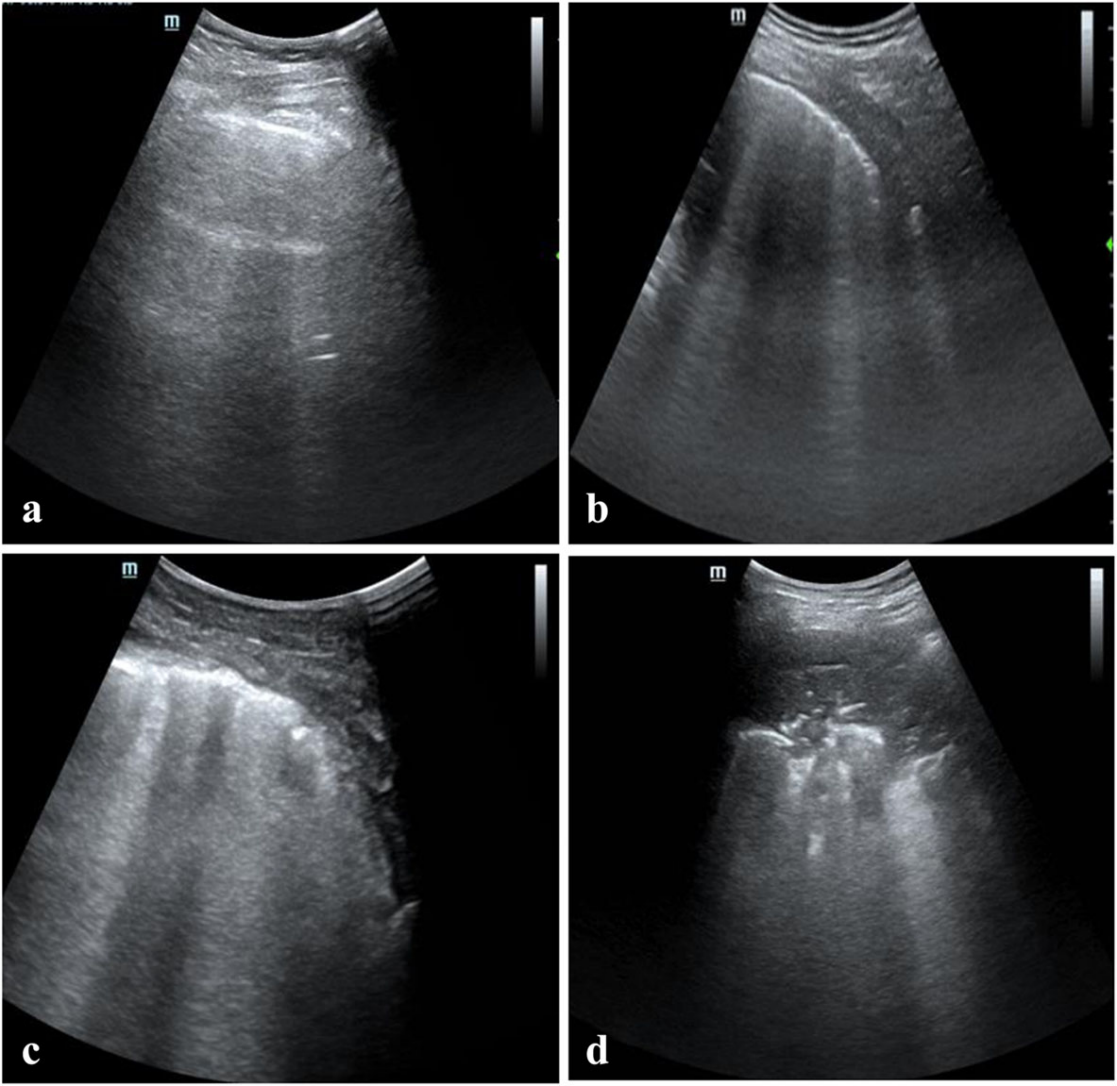
Lung ultrasound signs with different scores. **a** score 0, healthy lung, equidistant A-lines parallel to the sliding pleura; **b** score 1, moderate aeration loss, no fewer than 3 dispersive B lines originated from the pleura; **c** score 2, serious aeration loss, presence of coalescent B lines with irregular pleura; **d**, score 3, absolute aeration loss, subpleural consolidation. Xie et al. 2020 [2]
